# Proposed New Constitution and By-Laws

**Published:** 1905-04

**Authors:** 


### PROPOSED NEW CONSTITUTION AND BY-LAWS.

(Concluded from March issue of THE JOURNAL-RECORD OF MEDICINE.)

### CHAPTER V.—ELECTION OF OFFICERS.

SECTION 1. All elections shall be by ballot, and a majority of the votes cast shall be necessary to elect.

SEC. 2. The election of officers shall be the first order of business of the House of Delegates after the reading of the minutes on the morning of the last day of the General Session.

SEC. 3. The President of the Association shall be elected by ballot without a nomination, at one o'clock, P. M., on the last day of the meeting of the Association, and if there is no election on the first ballot, the three names receiving the highest number of ballots shall be voted on, the other names being dropped. If there is no election on second ballot, the two names receiving the highest number of ballots shall be voted on until election occurs.

SEC. 4. Any person known to have solicited votes for or sought any office within the gift of this Association shall be ineligible for any office for two years.

### CHAPTER VI.—DUTIES OF OFFICER.

SECTION 1. The President shall preside at all meetings of the Association and of the House of Delegates; shall appoint all committees not otherwise provided for; he shall deliver an annual address at such time as may be arranged, and perform such other duties as custom and parliamentary usage may require. He shall be the real head of the profession of the State during his term of office, and, as far as practicable, shall visit by appointment the various sections of the State and assist the Councilors in building up the county societies, and in making their work more practical and useful.

SEC. 2. The Vice-President shall assist the President in the discharge of his duties. In the event of the President's death, resignation or removal, the Council shall select one of the Vice-Presidents to succeed him.

SEC. 3. The Secretary and Treasurer shall give bond in the sum of $1,000. He shall demand and receive all funds due the Association, together with the bequests and donations. He shall pay money out of the Treasury only on a written order of the President; he shall subject his accounts to such examination as the House of Delegates may order, and he shall annually render an account of his doings and of the state of the funds in his hands.

SEC. 4. The Secretary and Treasurer shall attend the General Meetings of the Association and the meetings of the House of Delegates, and shall keep minutes of their respective proceedings in separate record books. He shall be *ex-officio* Secretary of the Council. He shall be custodian of all record books and papers belonging to the Association. He shall provide for the registration of the members and delegates at the Annual Sessions. He shall, with the co-operation of the secretaries of the component societies, keep a card-index register of all the legal practitioners of the State by counties, noting on each his status in relation to his county society, and, on request, shall transmit a copy of this list to the American Medical Association. He shall aid the Councilors in the organization and improvement of the county societies and in the extension of the power and usefulness of this Association. He shall conduct the official correspondence notifying members of meetings, officers of their election, and committees of their appointment and duties. He shall employ such assistants as may be ordered by the House of Delegates, and shall make an annual report to the House of Delegates. He shall supply each component society with the necessary blanks for making their annual reports; shall keep an account with the component societies, charging against each society its assessment and collect the same. Acting with the Committee on Scientific Work, he shall prepare and issue all programs. The amount of his salary shall be fixed by the House of Delegates.

CHAPTER VII.—COUNCIL.

SECTION 1. The Council shall meet on the day preceding the Annual Session, and daily during the Session, and at such other times as necessity may require, subject to the call of the chairman, or on petition of three Councilors. It shall meet on the last day of the Annual Session of the Association to organize and outline work for the ensuing year. It shall elect a chairman and a clerk, who, in the absence of the Secretary of the Association, shall keep a record of its proceedings. It shall, through its chairman, make an annual report to the House of Delegates.

SEC. 2. Each Councilor shall be organizer, peacemaker and censor for his district. He shall visit the counties in his district at least once a year for the purpose of organizing component societies where none exist; for inquiring into the condition of the profession, and for improving and increasing the zeal of the county societies and their members. He shall make an annual report of his work and of the condition of the profession of each county in his district at the Annual Session of the House of Delegates. The necessary traveling expenses incurred by such Councilor in the line of the duties herein imposed may be allowed by the House of Delegates on a proper itemized statement, but this shall not be construed to include his expense in attending the Annual Session of the Association.

SEC. 3. The Council shall be the board of censors of the Association. It shall consider all questions involving the rights and standing of members, whether in relation to other members, to the component societies, or to this Association. All questions of an ethical nature brought before the House of Delegates or the General Meeting shall be referred to the Council without discussion. It shall hear and decide all questions of discipline affecting the conduct of members or component societies on which an appeal is taken from the decision of an individual Councilor, and its decision in all such matters shall be final.

SEC. 4. In sparsely settled sections it shall have authority to organize the physicians of two or more counties into societies, to be suitably designated so as to distinguish them from district societies, and these societies, when organized and chartered, shall be entitled to all rights and privileges provided for component societies until such counties shall be organized separately.

SEC. 5. The Council shall provide for and superintend the publication and distribution of all proceedings, transactions and memoirs of the Association, and shall have authority to appoint an editor and such assistants as it deems necessary. All money received by the Council and its agents, resulting from the discharge of the duties assigned to them, must be paid to the Secretary and Treasurer of the Association. As the Finance Committee it shall annually audit the accounts of the Secretary and Treasurer and other agents of this Association and present a statement of the same in its annual report to the House of Delegates, which report shall also specify the character and cost of all the publications of the Association during the year, and the amount of all other property belonging to the Association under its control, with such suggestions as it may deem necessary. In the event of a vacancy in the office of the Secretary and Treasurer, the Council shall fill the vacancy until the next annual election.

### CHAPTER VIII.—COMMITTEES.

SECTION 1. The standing committees shall be as follows:

A Committee on Scientific Work.

A Committee on Public Policy and Legislation.

A Committee on Arrangement, and such other committees as may be necessary. Such committees shall be elected by the House of Delegates, unless otherwise provided.

SEC. 2. The Committee on Scientific Work shall consist of three members, of which the Secretary shall be one, and shall determine the character and scope of the scientific proceedings of the Association for each session, subject to the instructions of the House of Delegates. Thirty days previous to each Annual Session it shall prepare and issue a program announcing the order in which papers, discussions and other business shall be presented.

SEC. 3. The Committee on Public Policy and Legislation shall consist of three members and the President and Secretary. Under the direction of the House of Delegates it shall represent the Association in securing and enforcing legislation in the interest of public health and of scientific medicine. It shall keep in touch with professional and public opinion, shall endeavor to shape legislation so as to secure the best results for the whole people, and shall strive to organize professional influence so as to promote the general good of the community in local, State and national affairs and elections.

SEC. 4. The Committee of Arrangements shall be appointed by the component society in which the Annual Session is to be held. It shall provide suitable accommodations for the meeting-places of the Association and of the House of Delegates, and of their respective committees, and shall have general charge of all the arrangements. Its Chairman shall report an outline of the arrangements to the Secretary for publication in the program, and shall make additional announcements during the session as occasion may require.

CHAPTER IX.—COUNTY SOCIETIES.

SECTION. 1. All county societies now in affiliation with this Association or those which may hereafter be organized in this State, which have adopted principles of organization not in conflict with this Constitution and By-Laws, shall, on application, receive a charter from and become a component part of this Association.

SEC. 2. As rapidly as can be done after the adoption of this Constitution and By-Laws, a medical society shall be organized in every county in the State in which no component society exists, and charters shall be issued thereto.

SEC. 3. Charters shall be issued only upon approval of the Council and shall be signed by the President and Secretary of this Association. The House of Delegates shall have authority to revoke the charter of any component society whose actions are in conflict with the letter or spirit of this Constitution and By-Laws.

SEC. 4. Only one component medical society shall be chartered in any county except in counties containing one of our few largest cities. Where more than one county society exists, friendly overtures and concessions shall be made, with the aid of the Councilor for the District if necessary, and all of the members brought into one organization. In case of failure to unite, the Council shall decide what action shall be taken.

SEC. 5. Each county society shall judge of the qualification of its own members, but, as such societies are the only portals to this Association and to the American Medical Asssociation, every reputable and legally registered physician who does not practice or claim to practice, nor lend his support to, any exclusive system of medicine, shall be entitled to membership. Before a charter is issued to any county society, full and ample notice and opportunity shall be given to every such physician in the county to become a member.

SEC. 6. Any physician who may feel aggrieved by the action of the society of his county in refusing him membership, or in suspending or expelling him, shall have the right to appeal to the Council, and its decision shall be final.

SEC. 7. In hearing appeals the Council may admit oral or written evidence as in its judgment will best and most fairly present the facts, but in case of every appeal, both as a Board and as individual Councilors in district and county work, efforts at conciliation and compromise shall precede all such hearings.

SEC. 8. When a member in good standing in a component society moves to another county in this Sote, his name, on request, shall be transferred without cost to the roster of the county society into whose jurisdiction he moves.

SEC. 9. A physician living on ar near a county line may hold his membership in that county most convenient for him to attend, on permission of the society in whose jurisdiction he resides.

SEC. 10. Each component society shall have general direction of the affairs of the profession in its county, and its influence shall be constantly exerted for bettering the scientific, moral and material condition of every physician in the county; and systematic effort shall be made by each member, and by the society as a whole, to increase the membership until it embraces every qualified physician in the county.

SEC. 11. At some meeting in advance of the Annual Session of this Association, each county society shall elect a delegate or delegates to represent it in the House of Delegates of this Association, in the proportion of one delegate to each fifty members or fraction thereof, and the Secretary of the society shall send a list of such delegates to the Secretary of this Association at least ten days before the Annual Sessions.

SEC. 12. The Secretary of each component society shall keep a roster of its members and of the non-affiliated registered physicians of the county, in which shall be shown the full name, address, college, and date of graduation, date of license to practice in this State, and such other information as may be deemed necessary. In keeping such roster the Secretary shall note any changes in the personnel of the profession by death, or by removal to or from the county, and in making his annual report he shall be certain to account for every physician who has lived in the county during the year.

SEC. 13. The Secretary of each component society shall forward its assessment, together with its roster of officers and members, list of delegates, and list of non-affiliated physicians of the county to the Secretary of this Association each year thirty days before the Annual Session.

SEC. 14. Any county society which fails to pay its assessment, or make the report required, on or before April 1 of each year, shall be held as suspended, and none of its members or delegates shall be permitted to participate in any of the business or proceedings of the Association or of the House of Delegates until such requirements have been met.

### CHAPTER X.—MISCELLANEOUS.

SECTION 1. No address or paper before the Association, except those of the President and orators, shall occupy more than twenty minutes in its delivery; and no member shall speak longer than five minutes, nor more than once on any subject except by unanimous consent.

SEC. 2. All papers read before the Association or any of the Sections shall become its property. Each paper shall be deposited with the Secretary when read.

SEC. 3. The deliberations of this Association shall be governed by parliamentary usage as contained in Roberts' Rules of Order, when not in conflict with this Constitution and By-Laws.

SEC. 4. The Principles of Medical Ethics of the American Medical Association shall govern the conduct of members in their relations to each other and to the public, except as amended by the House of Delegates.

### CHAPTER XI.—AMENDMENTS.

These By-Laws may be amended at any Annual Session by a majority vote of all the delegates present at that session, after the amendment has laid on the table for one day.

---

AN exchange states that serum treatment has thus far failed to cure "summer complaint" in children. Less than a month after the death of his grandchild in 1900 Mr. John Rockefeller had put the machinery of science into motion to discover the cause of the disorder so fatal to infants commonly known as summer complaint. He announced an initial fund of $200,000 to be devoted to research, and asked Dr. William H. Welch, who is one of the most distinguished pathologists in the world, to conduct the inquiry. In 1902 it was announced that an antidysenteric serum had been discovered. Since that time twelve bacteriologists have been in charge of investigations in New York, Boston, Philadelphia and Baltimore. The second annual report of the board, just completed, says the serum is not a success. Almost half the children treated died, and in only a few eases has a noteworthy improvement followed its administration. Dr. Simon Flexner, of the University of Pennsylvania, who wrote this section of the report, declares that the figures indicate little of the gravity of the infection, since it is well known that the result in all forms of intestinal diseases depends upon the age and previous condition of the patient.

